# CRISPR‐S: an active interference element for a rapid and inexpensive selection of genome‐edited, transgene‐free rice plants

**DOI:** 10.1111/pbi.12788

**Published:** 2017-08-23

**Authors:** Hai‐Ping Lu, Song‐Mei Liu, Shou‐Ling Xu, Wu‐Yang Chen, Xin Zhou, Yuan‐Yuan Tan, Jian‐Zhong Huang, Qing‐Yao Shu

**Affiliations:** ^1^ National Key Laboratory of Rice Biology and Zhejiang Provincial Key Laboratory of Crop Germplasm Resources Institute of Crop Science Zhejiang University Hangzhou China; ^2^ Hubei Collaborative Innovation Center for the Grain Industry Jingzhou Hubei China; ^3^ Institute of Nuclear Agricultural Sciences Zhejiang University Hangzhou China

**Keywords:** Genome editing, T‐DNA free, RNAi

Dear Editor,

The CRISPR/Cas9‐based genome‐editing tool has been used in diverse applications related to plant research, including for crop improvement (Liu *et al*., 2017; Sun *et al*., 2016). Mutant plants may be generated *via* transient transformations or DNA‐free editing (Liang *et al*., 2017). However, plant genomes are often edited during the production of transgenic plants, in a process that involves the identification of targeted edits in regenerated T_0_ plants and the subsequent elimination of transgenes in T_1_ plants (Sun *et al*., 2016). Because T‐DNA (e.g. CRISPR/Cas9) is randomly inserted into plant genomes, it may be silent (e.g. when incorporated in heterochromatin) or is actively silenced; hence, theoretically only a portion of the T_0_ plants carry active transgenes, although high genome‐editing rates have been observed (Xie *et al*., 2015). Therefore, selecting transgene‐free plants with an edited genome may be a time‐consuming and costly part of genome‐editing projects. This is especially true for practical breeding programmes when many plants need to be produced and screened, considering that successful application is often genotype dependent (Zhu *et al*., 2017).

The RNA interference (RNAi) technique has already been used to decrease the abundance of unwanted grain ingredients or increase resistance to viral pathogens (Kamthan *et al*., 2015). Functional RNAi elements (e.g. hairpin RNAi, hpRNAi) can be detected in T_0_ plants because of their dominant nature. Therefore, we hypothesized that when an RNAi expression element is incorporated into a CRISPR/Cas9 vector, the activity and presence of the T‐DNA in the transgenic plants can be monitored based on RNAi. This would enable a PCR‐free, phenotype‐based identification of genome‐edited T_0_ plants, and a subsequent selection of transgene‐free T_1_ plants.

In rice, *CYP81A6* encodes a P450 cytochrome protein that confers resistance to bentazon. Silencing *CYP81A6* may render rice plants susceptible to the herbicide (Pan *et al*., 2006). We speculated that a *CYP81A6‐*hpRNAi may be a suitable marker for CRISPR/Cas9 expression. To produce a *CYP81A6‐*hpRNAi element, we first amplified a 300‐bp fragment from *CYP81A6* using forward (5′AGCTTAGCCATGGATAACGCCTAC3′) and reverse (5′AAGGTCACGTCGTGCTCGGTGAAGCACTC3′) primers. The fragment was then inserted into the pBSSK‐IN vector to form a hairpin structure, which was then introduced into a pCAMBIA‐1300 vector between the double 35S (*d35S*) promoter and the *Nos* terminator (*nos*). We inserted the *d35S‐hpRNAi‐nos* element into the HpaI site near the left border of a CRISPR/Cas9 vector, pHun4c12, to construct a new CRISPR/Cas9 vector, pHun4c12s (Figure 1a). To test the utility of pHun4c12s, we constructed another CRISPR/Cas9 expression vector, pHun4c12s‐*lct1*, to target *OsLCT1*, which encodes a Cd transporter (Uraguchi *et al*., 2011). Ninety‐six independent transgenic T_0_ plants were generated via *Agrobacterium tumefaciens*‐mediated transformation of *japonica* rice genotype Xidao #1 cells with pHun4c12s‐*lct1* and analysed further.

**Figure 1:**
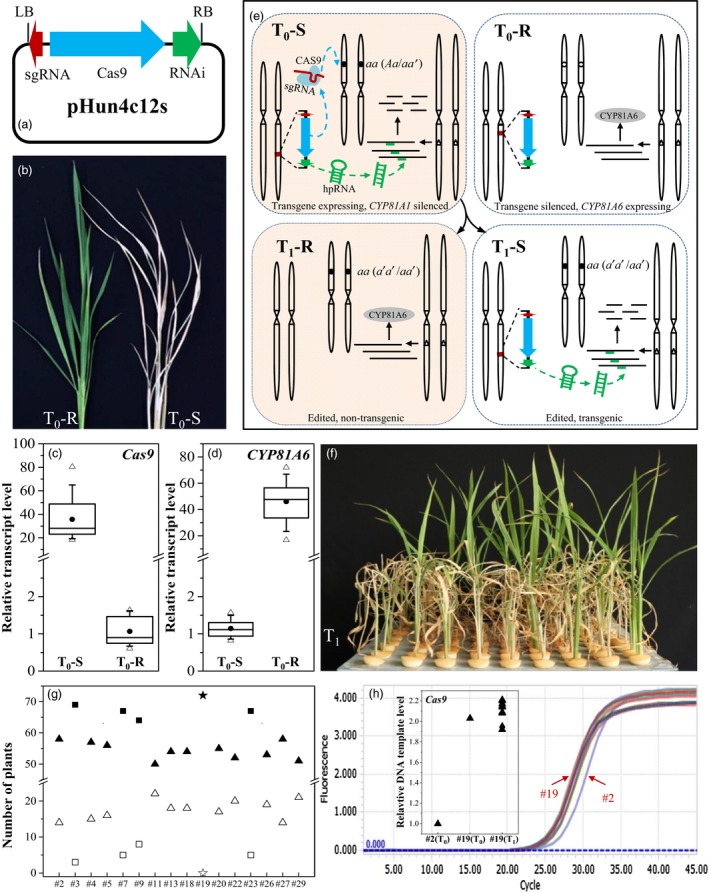
Production of genome‐edited, transgene‐free rice plants. (a) Diagram of a CRISPR‐S vector, pHun4c12s, derived from pHun4c12. (b) Bentazon‐resistant (*left,* T_0_‐R) and bentazon‐susceptible (*right,* T_0_‐S) tillers of transgenic T_0_ plants. (c) and (d) Box plot representations of *Cas9* (c) and *CYP81A6* (d) relative transcript levels of T_0_‐S and T_0_‐R plants. The values of one arbitrarily chosen T_0_‐R plant (c) and one T_0_‐S plant (d) were set as 1. For each box plot, the 10th, 25th, median, 75th and 90th percentiles of relative transcript levels were represented by horizontal lines (bottom to top), the average as filled circles, and the minimum and maximum indicated by empty triangles. (e) Illustration of the genetics of genome editing, bentazon susceptibility, and segregation of transgene and genome edits. In transgene‐expressing T_0_ plants, the target gene (empty circle) is edited by the CAS9 and sgRNA complex to introduce mutations (filled circle). Meanwhile, *CYP81A6‐hpRNAi* degrades *CYP81A6* transcripts, rendering plants susceptible to bentazon. In contrast, the genomes of T_0_ plants with a silenced transgene are not edited, and these plants remain resistant to bentazon. Transgene‐free (i.e. resistant to bentazon), genome‐edited T_1_‐R plants are produced by bentazon treatment, which kills transgenic T_1_‐S plants. (f) Symptoms of Line #2 T_1_ seedlings 1 week after a bentazon foliar spray (1000 mg/L). (g) Segregation of bentazon‐susceptible (filled) and bentazon‐resistant (empty) plants in 16 T_1_ lines. Lines fit the 3:1 or 16:1 ratios based on a chi‐square test are indicated with triangles or squares, respectively. Line #19 T_1_ plants are indicated with a filled star. (h) T‐DNA copy number in Line #19 plants assessed by a qRT‐PCR analysis of *Cas9*, with *Actin* as the internal standard, and the value of Line #2 T_0_ plant set as 1. *Actin* was used as the internal standard in qRT‐PCR analysis.

First, we tested the susceptibility of the T_0_ plants to bentazon using a foliar spray. Tillers of T_0_ plants were separated and grown as two subplants. One normally growing subplant of each T_0_ plant was treated with a bentazon solution (2000 mg/L) by spraying until droplets were visible on leaves. About 1 week later, 29 T_0_ subplants appeared to be susceptible and eventually died (T_0_‐S plants; Figure 1b). The other 67 T_0_ plants were not visibly affected (T_0_‐R plants).

Second, we sequenced the *OsLCT1* target region of the T_0_ plants. All 29 T_0_‐S plants were affected by homozygous (*aa*), biallelic (*aa′*) or heterozygous (*Aa*) mutations in the target region. Mutations were not detected in the remaining plants. Thus, bentazon susceptibility was 100% correlated with the targeted mutations in T_0_ plants.

Third, we analysed the abundance of *Cas9* and *CYP81A6* transcripts in T_0_‐R and T_0_‐S plants using a quantitative real‐time PCR (qRT‐PCR). Overall, the *Cas9* transcript was significantly more abundant in T_0_‐S plants than in T_0_‐R plants (Figure 1c). In contrast, the abundance of the *CYP81A6* transcript was lowest in the T_0_‐S plants (Figure 1d), suggesting that *CYP81A6‐hpRNAi* was more highly expressed in these plants. The expression of *CYP81A6‐hpRNAi* resulted in the degradation of *CYP81A6* transcripts *via* RNAi in transgene‐expressing, T_0_‐S plants. In contrast, the transgene‐silenced T_0_ plants remained resistant to bentazon (Figure 1e). These observations imply that *CYP81A6‐hpRNAi* enables the efficient selection of genome‐edited T_0_ plants.

Our system was also designed to simplify the selection of transgene‐free T_1_ plants. We grew 72 seedlings for each of 16 T_1_ lines derived from homozygous or biallelic *OsLCT1* mutant T_0_ plants treated them with 1000 mg/L bentazon in a foliar spray (approximately 100 mL/m^2^) at around the four‐leaf stage. About 4 days later, we observed that all Xidao #1 seedlings were growing normally, but some of the T_1_ seedlings started dehydrating and eventually died (Figure 1f). Furthermore, we proved that all bentazon‐susceptible plants were transgenic, while all bentazon‐resistant plants lacked T‐DNA. The segregation of T_1_ plants derived from a homozygous or biallelic genome‐edited T_0_ plant is presented in Figure 1e, in which the genome‐edited, transgene‐free plants are resistant to bentazon because the *CYP81A6‐hpRNAi* no longer exists. Meanwhile, transgenic plants are susceptible because the *CYP81A6* transcripts are degraded.

Transgenic plants produced by *A. tumefaciens*‐mediated transformation often carry one or two copies of T‐DNA (Collier *et al*., 2017). The segregation ratio observed for bentazon susceptibility was consistent with this fact in all T_1_ lines except for Line #19 (Figure 1g). All Line #19 T_1_ seedlings were susceptible to bentazon and died. Based on the 3:1 segregation ratio of its T_1_ population (Figure 1g), the Line #2 T_0_ plant was expected to have a single T‐DNA insertion and was therefore used as the control for the qRT‐PCR analysis. The Line #19 T_0_ plant and its T_1_ progenies appeared to have the same T‐DNA copy number, and they all had double the number of insertions of the Line #2 T_0_ plant (Figure 1h). This implies that T‐DNA copies were incorporated into sister chromosomes in Line #19 T_0_ plant. Otherwise, variability in the T‐DNA copy numbers among the T_1_ plants would have been detected.

To test the utility of CRISPR‐S for other genes, we constructed a pHun4c12s‐*frg* vector to target *OsBADH2* (Bradbury *et al*., 2005). We transformed nine rice genotypes and obtained 4–22 T_0_ plants for each genotype. All T_0_ plants susceptible to bentazon were confirmed to carry mutations. Similarly, transgene‐free, *OsBADH2*‐edited T_1_ plants were identified following a bentazon treatment of T_1_ seedlings.

The results of our proof‐of‐concept study revealed that the *Cas9* expression level could be indirectly estimated using a marker trait generated by an RNAi element incorporated into the CRISPR/Cas9 expression vector. This new CRISPR/Cas9 system provides a relatively simple way of identifying and eliminating T_0_ plants in which the genome has not been edited. More importantly, our method enables the selection of transgene‐free T_1_ plants, with almost no cost, confirming the value of this system.

Antibiotic or herbicide resistance genes in binary vectors are important for selecting transgenic plants. However, they cannot be directly used to select transgene‐free T_1_ plants because they would kill the plants. Seed‐localized fluorescent reporters have been used to discriminate between transgenic and nontransgenic seeds in a few plant species. Unfortunately, they are unsuitable for species that produce seeds with hulls or glumes such as rice. Although our system has been demonstrated in rice, it may be possible to develop similar systems in other plant species. Highly conserved homologs of *CYP81A6* have been detected in monocots. Thus, the bentazon‐susceptibility trait may also be used in these plant species. Furthermore, other suitable marker traits can be generated by down‐regulating the expression of other genes to introduce visible morphological changes to leaf shape and colour, among other characteristics.

## Funding

This work was supported by the Zhejiang Provincial S & T Project on Breeding of Agricultural (Food) Crops (grant no. 2016C02050‐2).

## Author Contributions

Q.‐Y. S., H.‐P. L. and J.‐Z. H designed the experiments, analysed the data and wrote the manuscript; H.‐P. L. conducted the experiments with the assistance of S.‐M. L., S.‐L. X., W.‐Y. C., X. Z. and Y.‐Y. T.
